# Draft genome of *Bacillus subtilis* sp. strain UAMC isolated from agricultural soil with historical use of pesticides in Argentina

**DOI:** 10.1128/mra.00188-24

**Published:** 2024-06-11

**Authors:** Adriana Casanova, Diego A. Esquivel-Hernández, Dolores Reyes-Duarte, Sergio Hernández, Irmene Ortíz

**Affiliations:** 1 Posgrado en Ciencias Naturales e Ingeniería. Universidad Autónoma Metropolitana-Cuajimalpa, México City, Mexico; 2 Departamento de Procesos y Tecnología, Universidad Autónoma Metropolitana-Cuajimalpa, México City, Mexico; University of Southern California, Los Angeles, California, USA

**Keywords:** polluted soils, pesticides, *Bacillus subtilis*, genomics

## Abstract

To understand microbial metabolism in horticultural soils exposed to pesticides, genome sequencing of *Bacillus subtilis* sp. strain UAMC was performed. A total of 7,892 genes distributed across 40 contigs were identified. Among these, those related to the degradation of endosulfan such as FMNH_2_ monooxygenase, or cytochrome p450 stand out.

## ANNOUNCEMENT

The environmental and health problems caused by the excessive use of pesticides, such as endosulfan, have driven environmental remediation strategies (1, 2). In this way, *Bacillus subtilis* is known for its potential use in soil and water bioremediation and its ability to enhance plant growth (3, 4).

In this study, *Bacillus subtilis* sp. strain UAMC was isolated from the “Cinturón hortícola platense” in La Plata, Argentina (34° 51′ 23.897″ S, 58° 16′ 19.198″ O) as previously reported (3). The bacterial culture was grown in LB medium at 30°C and 150 rpm for genomic analysis. DNA extraction was performed using the Presto soil DNA extraction kit (SLD050, Geneaid) following the manufacturer’s instructions (3). The purity of the genomic was checked by absorbance measured by spectrophotometry in a Nanodrop 2000 with a 1.94 ratio between OD260/OD280, indicating no significant contamination. Illumina Nextera XT paired-end library was prepared. Whole-genome sequencing (WGS) was conducted using a Miseq v2 system (Illumina) Micro-run with 300 cycles, generating 256,892 paired-end reads with an average length of 150 bp. The raw sequence was processed using the Kbase platform (https://kbase.us/n/171478/26/) (5). During the analysis, default parameters were employed for all software, unless explicitly stated otherwise. Read quality assessment was conducted utilizing FastQC version 0.12.1 (6, 7) (where 256,196 high-quality reads were reported from 38 Mbp, with an average GC content of 43%), with adaptor sequences unique to the Nextera DNA library being trimmed *via* Trimmomatic version 0.36 (8). *De novo* assembly conducted with SPAdes version 3.15.3 yielded 40 contigs (9). The draft genome sequence comprised a total length of 4,100,810 base pairs, exhibiting an N50 value of 1,027,590 base pairs and a GC content of 43.53%. Genome assembly metrics were calculated using QUAST version 4.4 (10). Genome coverage (9-fold) was calculated using BEDTools (11). Contig sizes ranged from 506 bp to 1,028,498 bp. Assessment for completeness and contamination was performed using CheckM version 1.0.18, indicating a 99.59% completion rate with 0.23% contamination (12). *Bacillus subtilis* sp. strain UAMC was most closely related to *Bacillus subtilis* subsp. subtilis str. 168, with an average nucleotide identity of 98.35% as determined by ANItools (13). The strain was taxonomically identified through GTDB-Tk v1.7.0 (14). The genome sequence of the *Bacillus subtilis* sp. strain UAMC underwent initial annotation *via* Prokka version 1.14.5, which identified a total of 4,099 protein-coding genes within the genome (https://bit.ly/441RZjV) (15). Subsequently, functional annotation was conducted using Rapid Annotations using Subsystems Technology (RAST) version 1.073, wherein homologous genes with established taxonomic affiliations were identified, assigning 4,320 coding genes and confirming the taxonomy of *Bacillus subtilis* sp. strain UAMC (https://bit.ly/3xD7zGs) (16). Several genes were identified, some particularly noteworthy were FMNH_2_-dependent alkanesulfonate monooxygenase (EC 1.14.14.5), gluconolactonase (EC 3.1.1.17), hydrolase of the alpha/beta fold family, and cytochrome P450 CypA possibly involved in the biodegradation of endosulfan (17). The genomic data from this *Bacillus subtilis* sp. strain UAMC will enhance our comprehension of how it metabolizes endosulfan derivatives, thereby expanding our knowledge of genes and their enzymes potentially related to organochlorine pesticide biotransformation.

## Data Availability

The assembly and its raw sequencing reads are available in the National Center for Biotechnology Information under the accession number PRJEB72839.
